# Systematic differences of non-invasive dominant frequency estimation compared to invasive dominant frequency estimation in atrial fibrillation

**DOI:** 10.1016/j.compbiomed.2018.11.017

**Published:** 2019-01

**Authors:** Frederique J. Vanheusden, Gavin S. Chu, Xin Li, João Salinet, Tiago P. Almeida, Nawshin Dastagir, Peter J. Stafford, G. André Ng, Fernando S. Schlindwein

**Affiliations:** a Department of Engineering, School of Science & Technology, Clifton Campus, Nottingham Trent University, Clifton Lane, Nottingham, NG11 8NS, United Kingdom; b Department of Engineering, University of Leicester, University Road, Leicester, LE1 7RH, United Kingdom; c Department of Cardiovascular Sciences, University of Leicester, Clinical Sciences Wing, Glenfield General Hospital, Groby Road, Leicester, LE3 9QP, United Kingdom; d University Hospitals of Leicester NHS Trust, Glenfield General Hospital, Groby Road, Leicester, LE3 9QP, United Kingdom; e Biomedical Engineering, Centre for Engineering, Modelling and Applied Social Sciences, Federal University of ABC, Alameda da Universidade, Anchieta, São Bernardo do Campo, SP, 09606-045, Brazil; f Instituto Tecnológico da Aeronáutica (ITA), Praça Marechal Eduardo Gomes 50, Vila das Acacias, São José dos Campos, SP, 12228-900, Brazil; g National Institute for Health Research Leicester Cardiovascular Biomedical Research Centre, Leicester, United Kingdom

**Keywords:** Atrial fibrillation, Body surface mapping, Biomedical signal processing, Cardiology, Dominant frequency, Electrocardiography, Non-contact mapping, Volume conductor

## Abstract

Non-invasive analysis of atrial fibrillation (AF) using body surface mapping (BSM) has gained significant interest, with attempts at interpreting atrial spectro-temporal parameters from body surface signals. As these body surface signals could be affected by properties of the torso volume conductor, this interpretation is not always straightforward. This paper highlights the volume conductor effects and influences of the algorithm parameters for identifying the dominant frequency (DF) from cardiac signals collected simultaneously on the torso and atrial surface. Bi-atrial virtual electrograms (VEGMs) and BSMs were recorded simultaneously for 5 min from 10 patients undergoing ablation for persistent AF. Frequency analysis was performed on 4 s segments. DF was defined as the frequency with highest power between 4 and 10 Hz with and without applying organization index (OI) thresholds. The volume conductor effect was assessed by analyzing the highest DF (HDF) difference of each VEGM HDF against its BSM counterpart. Significant differences in HDF values between intra-cardiac and torso signals could be observed, independent of OI threshold. This difference increases with increasing endocardial HDF (BSM-VEGM median difference from −0.13 Hz for VEGM HDF at 6.25 ± 0.25 Hz to −4.24 Hz at 9.75 ± 0.25 Hz), thereby confirming the theory of the volume conductor effect in real-life situations. Applying an OI threshold strongly affected the BSM HDF area size and location and atrial HDF area location. These results suggest that volume conductor and measurement algorithm effects must be considered for appropriate clinical interpretation.

## Highlights

• Dominant frequency behavior of body surface signals and within both atria is compared simultaneously.
• Body surface dominant frequencies are consistently lower than on the atria.
• This behavior validates the theory of the volume conductor effect using real-world experimental data.
• OI thresholding can significantly affect dominant frequency value and location.
• DF mapping with suitable OI thresholding may improve specificity.

## 1 Introduction

Non-invasive analysis of atrial fibrillation (AF) using body surface maps (BSMs) to locate AF drivers has become increasingly popular in recent years [[1], [2], [3], [4]], motivated by the reduced costs and risks compared to electrophysiological (EP) studies, and the development of accurate systems for inverse problem analysis of atrial activity [5,6]. With the advent of new metrics for identifying drivers of AF [[7], [8], [9]], establishing methods for directly correlating these metrics as measured on the body surface to AF sources has gained significant interest [10,11]. This area of research can be seen as a step between direct (invasive) measurement of AF behavior and the non-invasive estimation of AF sources using inverse problem analysis. This type of analysis can find a clinical niche in both (early) diagnosis of AF and follow-up; it could give clinicians the possibility to optimize treatment of AF compared to standard 12-lead temporal ECG analysis, without the need of performing invasive studies or the need for additional imaging for AF source estimation using inverse procedures [3].

Several issues need to be addressed for an appropriate interpretation of body surface measurements and its relation to cardiac electrophysiology. In particular, basic aspects of the torso volume conductor and how they affect the interpretation of BSM need to be taken into account [12]. The exact electrical properties of tissues and organs, and therefore their effects on how the cardiac electrical signal ‘travels’ from the heart to the torso surface, are mostly patient-specific and can even be influenced by environmental changes [[13], [14], [15]]. Some simulation studies have suggested that the volume conductor can be simplified to a low-pass spatial filter [16,17]. BSMs can, therefore, be considered blurred images of cardiac signals, with body surface signals being weighted contributions of activity over the entire atrium [18].

Methodologically, current algorithms to analyze AF sources invasively, such as complex fractionated atrial electrograms (CFAEs), phase singularities, and highest dominant frequency (HDF) have not been standardized, leading to conflicting results across clinical studies [[19], [20], [21], [22]]. An elaborate intuition on how to derive and interpret HDFs from endocardial data has been presented by Ng and Goldberger in a series of publications [20,23,24]. Further recommendations on deriving frequency spectra using Welch periodograms [4] and optimal pre-processing steps for unipolar electrograms were recently presented [25]. Following these recommendations, it was shown that dominant frequency (DF) analysis could be performed reliably on both unipolar and bipolar endocardial signals [23]. An extrapolation of these results to clinical BSMs is, however, lacking.

This paper contributes to the current knowledge of AF assessment from BSMs in two ways. It will use the assessment of HDF as a starting point, as this AF feature can be derived straightforwardly from the cardiac signal by spectral (Fourier) analysis, and methodological aspects on its interpretation for endocardial data have been thoroughly investigated. First, it will extend the work on interpreting DF and HDF on BSMs by analyzing how simple changes in DF stability thresholds affect body surface and atrial DF maps. It will make use of the organization index (OI) for assessing DF stability. Second, it will test and validate the volume conductor effect by comparing HDF values measured simultaneously at the endocardial surface and at the body surface.

## 2 Methods

### 2.1 Dataset

The study involved 10 male subjects (median age 58, range 36–76) referred to our institution for first-time ablation of persistent AF (persAF). All subjects were in AF at the start of the procedure. Procedure duration was 6.5 (5–8) hours. Study approval was obtained from the local ethics committee and all procedures were performed with full informed consent.

### 2.2 Electrophysiological (EP) study

**Fig. 1:**
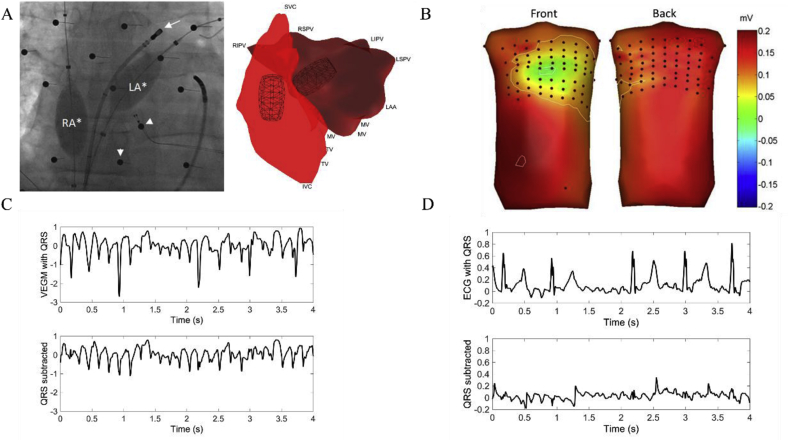
A. Intra-procedural fluoroscopy showing the presence of left atrial (LA) and right atrial (RA) non-contact arrays (white asterisks), Body Surface Mapping (BSM) electrodes (white arrow heads) and ablation catheter (white arrow); B. Overview of BSM setup during atrial fibrillation (AF). Electrodes positions are indicated with black dots. Interpolation was performed using the Surface Laplacian method [28]; C. Example of virtual electrogram data during AF before (top) and after (bottom) QRST subtraction; D. BSM data from the same time window as in C, before (top) and after QRS (bottom) subtraction.

Prior to the procedure, all anti-arrhythmic drugs were stopped for at least 5 half-lives, except for amiodarone. During the procedure, subjects were anticoagulated with heparin to maintain an activated clotting time above 300 s. Subjects were under general anesthesia during the entire procedure. For all subjects, bi-atrial 3-dimensional electro-anatomical mapping (3D EAM) was performed. First, a non-contact multi-electrode array (EnSite, St. Jude Medical, St. Paul, MN, USA) and a conventional deflectable ablation catheter were deployed into the right atrium (RA) with an anchoring point in the superior vena cava (SVC). Similarly, an EnSite array was positioned *trans*-septally into the left atrium (LA) with an anchoring point at the left upper pulmonary vein (LUPV). The distance between the balloon and the endocardial wall of both atria was kept below 4 cm to allow good quality reconstruction using the inverse procedure [26]. Further to this, a 7F coronary sinus (CS) catheter (Inquiry, St. Jude Medical) was deployed from the left femoral vein to support pacing protocols. A fluoroscopic image of the setup with both balloons in place is shown in Fig. 1A (left). Detailed atrial geometries were reconstructed with EAM software using the ablation catheter and anatomical landmarks were annotated (Fig. 1A, right). After mapping, both arrays were kept in position to avoid distortion of the reconstructed isopotential maps [27]. Additional ECG leads were positioned at the right arm, left arm and left leg to collect derivations I, II and III of the ECG.

Body surface signals were measured using a commercially available system (ActiveTwo, BioSemi, Amsterdam, The Netherlands) consisting of 131 electrodes (64 anterior, 64 posterior and 3 WCT electrodes). As the project focused on the analysis of atrial signals, a horizontal BSM setup (Fig. 1b) was developed to maximize electrode density around the atrial regions [29]. BSM, ECG and Virtual Electrogram (VEGM) data were collected simultaneously for 5 min before ablation. A CS pacing protocol was set up at the beginning of each measurement to help time alignment of data.

Atrial VEGM signals are atrial endocardial signals estimated from non-contact catheter electrodes placed inside the atrial cavity. The estimation is derived using an inverse solution (see Ref. [30] for details). The benefit of obtaining VEGMs from an inverse solution is that a panoramic view of the entire atrial endocardium can be obtained at any moment, whereas contact catheters only allow sequential measurements of a constrained area [26]. A limitation is that the accuracy of the estimated VEGMs is dependent on the inverse solution algorithm [18,31]. For the EnSite system used in this study, the non-contact balloon catheter contains 64 electrodes, from which 2048 VEGMs can be estimated and exported. The inverse solution is based on a potential field transfer matrix to estimate the potential field at a surface close to the source generating the (virtual) atrial electrograms from a distant potential (the location of balloon catheter) [18].

### 2.3 AF signal processing

VEGM data were extracted with the EnSite default band-pass filter set between 1 and 100 Hz for offline analysis using MATLAB (R2013b, Mathworks Inc., Natick, MA, USA), along with raw BSM data extracted using EEGLAB [32]. The quality of the BSM data was verified after zero-referencing and band-pass filtering between 2 and 50 Hz using a moving average filter [33]. After performing a Fast Fourier Transform (FFT), signals with a 50 Hz (mains in UK) peak power higher than 0.5% of the total spectral content were considered noisy and discarded [10]. Further evaluation of signal quality was assessed visually both on a signal-by-signal basis and after projection onto a standard 3D torso model [34]. After this evaluation, a minimum of 120 electrodes (124 ± 2, mean ± standard deviation) remained available for further analysis for each subject.

As BSM data were recorded at a different sampling rate (2048 Hz) compared to the EnSite system (1200 Hz and 2034.5 Hz for the RA and LA, respectively), data were resampled at 512 Hz and semi-automatically aligned at the R-peak of the fifth QRS complex after the pacing stimulus protocol. As a reference, ECG lead I collected for both the EnSite and BSM systems was used. The manually identified starting point for alignment was then localized automatically on all other signals and visually verified. To remove far-field (ventricular) activity, QRS-T cancellation was performed as described previously [21], leaving only the ‘clean’ atrial signal (Fig. 1C and D).

### 2.4 Dominant frequency (DF) analysis

For the purpose of evaluating the volume conductor effect, attention was given to the frequency analysis of the atrial VEGM and BSM signals. Frequency spectra were generated by applying Welch's method on 4 s windows (50% overlap, Hamming window). Zero-padding was applied to improve visualization of peaks (0.05 Hz bin-width) [21]. For each VEGM and BSM signal, the DF in a particular window was identified as the frequency with maximum power in the interval between 4 and 10 Hz in this window. This interval was chosen as it provides relevant information regarding AF electrophysiology [35].

Since previous research has suggested that HDF sites could represent AF drivers [7], HDFs were identified for each window for both BSM and VEGM signals. In this work, areas hosting the HDF on the atrium during a 4 s window were defined as those RA and LA VEGM signals whose DF value was within the maximum DF-0.25 Hz range measured over all 2048 signals in that particular window. Similarly, HDF values for each window on the BSM were defined as the BSM signals which expressed the maximum DF value or the maximum DF-0.25 Hz for that window.

A problem in the identification of the (highest) DF based on the frequency with the highest power within a spectrum lies in the possibility of detecting harmonics of the DF [20]. To determine the likelihood of HDF values to be genuine, an F-test was applied to analyze the significance of the HDF power compared to the power of neighboring frequencies [36]. For each HDF, an F-ratio was calculated by taking the ratio of power at the HDF frequency over the average power of the next 10 frequencies above and 10 frequencies below the HDF frequency. This F-ratio can then be tested for significance using an F-test with 2 and 2(F-1) degrees of freedom, where F is the number of frequencies included in the analysis (in this case 21, including the HDF and its 20 neighboring frequencies). The HDF peak was considered significant if the F-ratio was above the critical value of this F-test (3.23). If the HDF peak was not significant, the frequency with the highest power was determined in a frequency range between 1 Hz and the original HDF (to determine if the HDF was a harmonic of the cardiac cycle rate) and a frequency range between 4 Hz and the original HDF (to determine if the HDF was a harmonic of a lower frequency AF driver). If the HDF was identified as a harmonic, that window was excluded from further analysis.

For assessing the effect of including a HDF organization threshold, OI maps were generated. OI was defined as the ratio of the area of the DF and its harmonics up to 20 Hz, and the total area of the power spectrum [37]. For a signal in the frequency domain Y [f]:

(1) $$OI=\frac{\sum_{f=f_{DF}-k}^{f_{DF}+k}Y\left[f\right]+\sum_{n=1}^{N}\sum_{f=h_{n}-k}^{h_{n}+k}Y\left[f\right]}{\sum_{f=f_{l}}^{f_{h}}Y\left[f\right]}$$

where *h*_*n*_ are the harmonic peaks of the DF, *k* a DF width threshold (set at 0.375 Hz [38]), and *N* is the total number of harmonic peaks within a certain frequency band (in this case *f*_*l*_ = 0 and *f*_*h*_ = 20 Hz). DFs were defined for OI thresholds between 0 and 1 (steps of 0.1).

We investigated the effect of altering pre-processing steps on extracting HDFs as proposed by Li and colleagues [25], namely filtering the VEGMs between 20 and 100 Hz and rectifying these filtered signals. We examined the effect of estimating the AF spectral content using Welch's method and the FFT method. Besides this, the effect of averaging DFs over four consecutive windows was investigated [24]. Varying these preprocessing steps did not significantly alter the volume conductor effect observed between VEGM HDF and BSM HDF values. Results for these settings are therefore not further discussed in this paper. It is noteworthy to mention, however, that the stability of HDF values (based on organization index (OI) measurements) was higher when estimating spectral content using Welch's compared to the FFT method.

### 2.5 Comparing intracardiac and body surface measures of highest dominant frequency

**Fig. 2:**
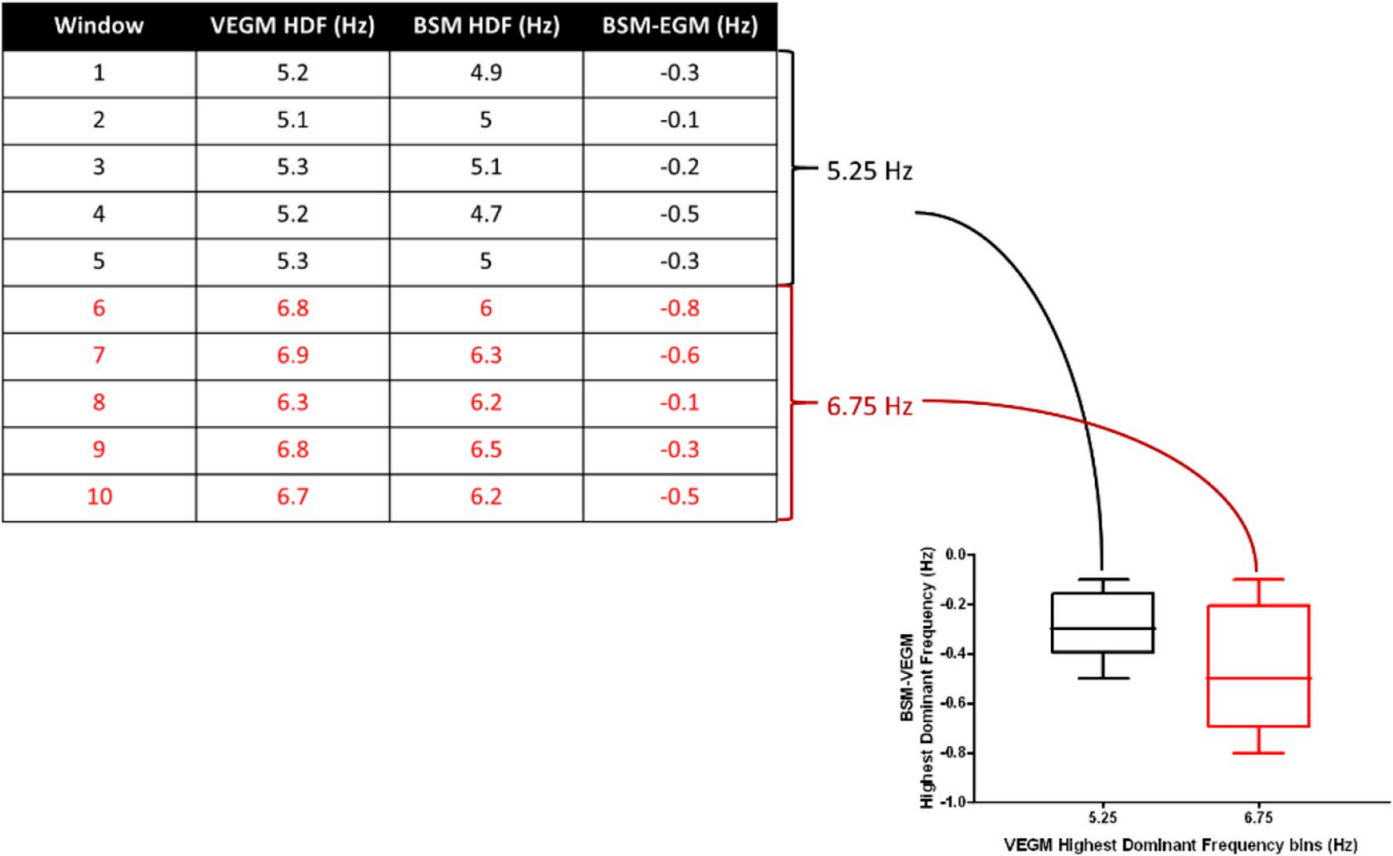
Example of Highest DF (HDF) binning for a hypothetical dataset. For each window, the HDF for the VEGMs is found. VEGM HDF values within a 0.5 Hz range are combined into a single HDF bin. The difference in HDF values between the BSM and VEGM data for all windows within one HDF bin are then collected and their distribution is represented as a boxplot. The median values of these boxplots were used to determine a correlation between VEGM HDFs and BSM HDFs.

Multiple previous studies have indicated the value of HDF sites in locating AF drivers [7,21,[38], [39], [40]]. In the present study, the HDF value was identified for every 4 s window in both 5-min BSM and VEGM datasets. HDF areas (intra-cardiac or torso geometry nodes) were defined in each 4 s window as those whose DF value lay within 0.25 Hz of the HDF for the given window. As intra-cardiac data would be expected to represent the gold standard measurement, in order to assess the precision of the BSM HDF, the difference in the HDF between BSM and VEGM data was calculated (BSM HDF – VEGM HDF) for each 4 s window separately and aggregated across all 9 patients. For visualization purposes, VEGM HDF values were then binned in groups of 0.5 Hz and correlated against their corresponding difference values. A schematic overview of this procedure is given in Fig. 2.

### 2.6 Assessing HDF significance using OI

A high OI is indicative of the dominance of the DF peak within the spectrum to which it pertains [[41], [42], [43]], and may be associated with clinically relevant AF sources [7,38,44,45]. To investigate its relevance in BSM, OI thresholds were set between 0 and 1 (steps of 0.1), and BSM HDF and VEGM HDF were defined as before but with the additional constraint that a node hosting HDF could only be defined if its OI exceeded the predefined threshold. This more specific method was applied to both BSM and VEGM data to investigate whether nodes of HDF could still be detected in each 4 s window. The effect on the precision of BSM HDF (as described above) was also assessed.

### 2.7 Statistics

Statistical analysis was performed with Prism 7 (GraphPad Software, Inc., La Jolla, California, USA). All statistical comparisons were analyzed at an alpha level of 0.05. Parameters were non-normally distributed according to the Shapiro-Wilk test, and were therefore represented by their median and interquartile range. Comparisons of metrics on BSM and VEGM signals were performed using a Wilcoxon matched-pairs signed rank test. Significance of the difference in area covered over all windows was also investigated (Wilcoxon matched-pair signed rank test). Optimization of curve fitting was achieved by minimization of non-linear least squares using the Levenberg-Marquardt algorithm.

## 3 Results

Data from one subject (patient 10) were excluded due to strong artefacts in the right atrial VEGMs. Further analysis was performed on the remaining 9 participants. A total of 1322 windows were available for HDF analysis on both VEGM and BSM data. After removing windows in which either VEGM or BSM data showed the potential of having detected a harmonic as the HDF, 1002 windows (75.80%) remained for further analysis.

**Fig. 3:**
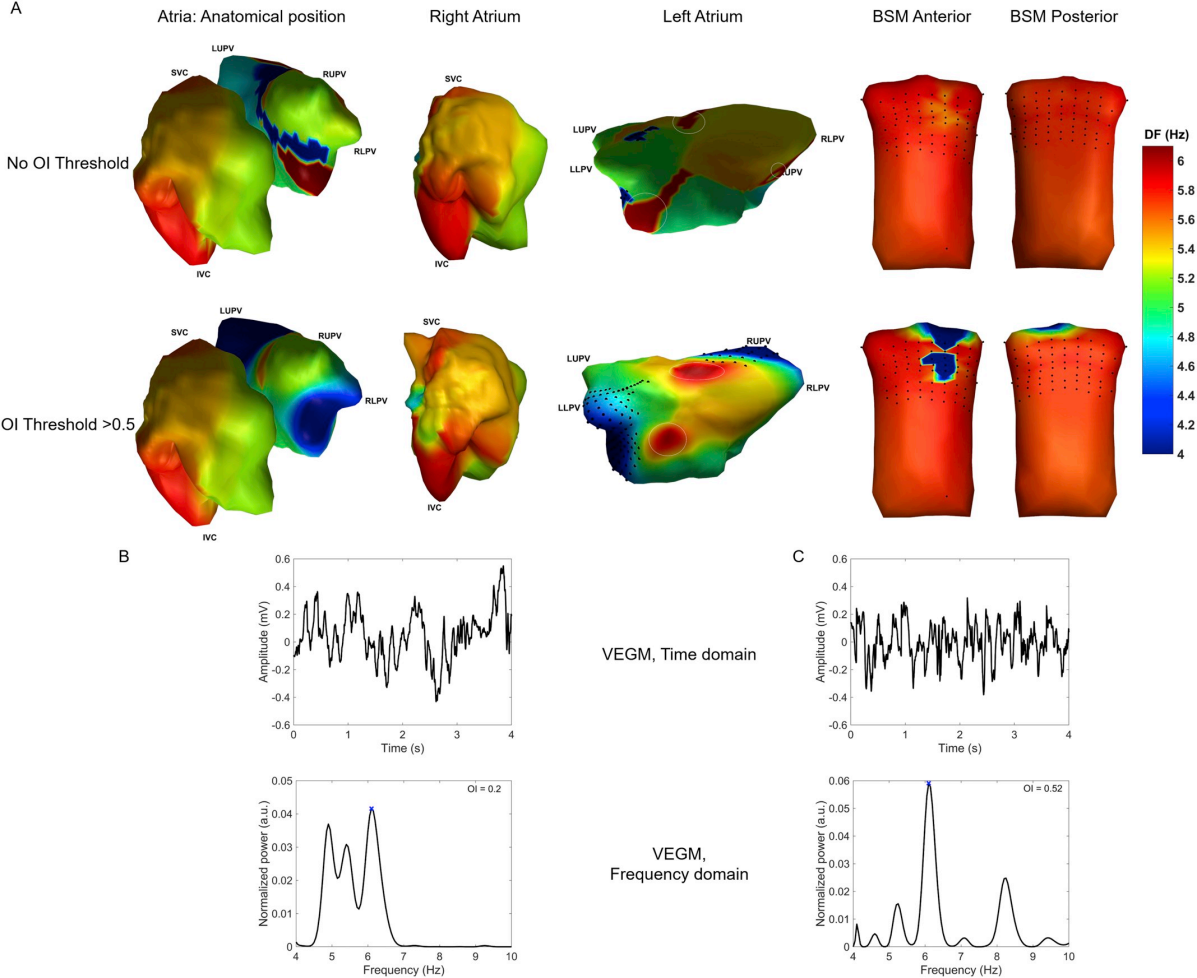
Example of a DF window without OI threshold, and when applying an OI threshold of 0.5. A. DF maps for the right and left atrium and body surface. Black dots on the left atrium indicate positions where no HDF could be determined. Black dots on the BSM indicate electrode locations. Colors at locations where no HDF was measured or identified are interpolated using the surface Laplacian for visualization purposes [28]. Areas of HDF on the atrium are indicated (white ellipses); B. VEGM signal and frequency spectrum for an HDF node from the window visualized on the DF maps. C. VEGM signal and frequency spectrum for a HDF node after OI thresholding (OI > 0.5). HDF area and location have both changed in the left atrium.

Fig. 3 gives an example of a DF map for the LA and RA and BSM for one 4 s window for OI thresholds of 0 and 0.5, respectively The OI threshold of 0.5 was chosen as it sits halfway between the OI extreme values (0–1). When applying an OI threshold, the ability to define a HDF for a specific window means that, for at least one of the electrodes, a DF could be observed with an OI above this threshold. The HDF was then considered to be the maximum DF over the electrodes for which a DF was observed.

Results indicate that the implementation of an OI threshold can significantly affect the representation of the DF and therefore modify the HDF value and location of the HDF areas (Table 1, Table 2). It can be observed that the percentage of nodes per window hosting HDF varies, with some subjects showing the HDF area enlarging whilst other subjects show the area shrinking after thresholding. On the body surface (Table 1), a window-by-window comparison showed that the change in HDF area after OI thresholding is significantly different for most subjects. OI thresholding can also identify new HDF-hosting locations. For example in subject 3, HDF location was maintained in less than half (46.06%) of the body surface nodes after applying an OI > 0.5 threshold, whilst on average 23.40% new nodes are identified in each window. On the other hand, subject 4 shows higher stability of its original HDF nodes, with on average 80.64% HDF nodes being maintained after OI thresholding.

**Table 1:** The effect of OI thresholding on HDF detection and localization with BSM data. Results are expressed as the proportion of valid nodes per 4s window on the body surface (median; interquartile range).

Subject	Number of windows analyzed (pairwise analysis)	% of BSM nodes hosting HDF when threshold OI = 0	% of BSM nodes hosting HDF when applying threshold OI > 0.5	p	% of all BSM HDF nodes continuing to host HDF after applying OI threshold per window	% of BSM HDF nodes when threshold OI > 0.5 which were not present when threshold OI = 0
1	74	12.08; 19.17	6.04; 13.27	**<0.001**	30.93; 37.41	22.23; 41.15
2	88	10.34; 19.54	10.57; 20.09	**<0.001**	60.13; 39.77	11.37; 30.76
3	95	5.09; 6.51	4.14; 5.69	**<0.001**	46.06; 40.75	23.40; 41.34
4	104	32.09; 38.08	34.08; 37.41	0.87	80.64; 35.63	13.71; 33.87
5	98	8.82; 13.60	8.36; 14.94	**<0.001**	44.29; 38.71	23.32; 41.72
6	62	5.41; 9.41	4.28; 10.00	**<0.001**	28.06; 37.43	29.58; 45.16
7	93	13.47; 20.70	14.23; 21.41	**0.004**	53.96; 39.21	18.00; 37.51
8	93	4.57; 9.37	5.32; 8.91	0.75	31.87; 39.37	42.39; 48.99
9	88	7.75; 11.81	6.36; 10.71	**<0.001**	54.73; 39.69	8.12; 26.86

**Table 2:** The effect of OI thresholding on HDF detection and localization with VEGM data. Results are expressed as the proportion of valid nodes per 4s window on the body surface (median; interquartile range). Significance of the difference in area covered over all windows is indicated (Wilcoxon matched-pair signed rank test, alpha = 0.05).

Subject	Number of windows analyzed (pairwise analysis)	% of VEGM nodes hosting HDF when threshold OI = 0	% of VEGM nodes hosting HDF when applying threshold OI > 0.5	p	% of BSM HDF nodes continuing to host HDF after applying OI threshold per window	% of VEGM HDF nodes when threshold OI > 0.5 which were not present when threshold OI = 0
1	74	2.12; 2.84	3.69; 6.41	**0.003**	0; 29.34	100; 46.52
2	88	3.17; 3.52	3.22; 3.62	0.41	27.61; 53.77	56.59; 94.70
3	95	3.13; 4.58	3.50; 4.57	0.96	28.68; 50.46	59.42; 88.60
4	104	4.96; 7.98	4.08; 6.27	**0.004**	15.08; 36.41	76.55; 94.59
5	98	2.91; 3.68	2.75; 3.51	0.10	0; 34.89	100; 81.71
6	62	2.59; 4.15	3.52; 4.15	0.25	0; 33.30	100; 71.24
7	93	4.70; 8.34	6.89; 10.1	0.70	60.72; 63.26	18.63; 87.2
8	93	4.00; 5.59	4.35; 6.21	0.42	22.36; 53.68	74.11; 98.06
9	88	2.99; 4.29	2.75; 3.66	0.27	0; 29.9	100; 81.63

For the VEGM signals (Table 2), only two subjects (1 and 4) show a significant difference in HDF area after OI thresholding. However, for all subjects, the location of the HDF nodes changes, as shown by the median percentage of nodes per window unaffected by OI thresholding being less than 50% for all subjects except subject 7 (60.72%).

### 3.1 Comparison of VEGM and BSM HDF values

**Fig. 4:**
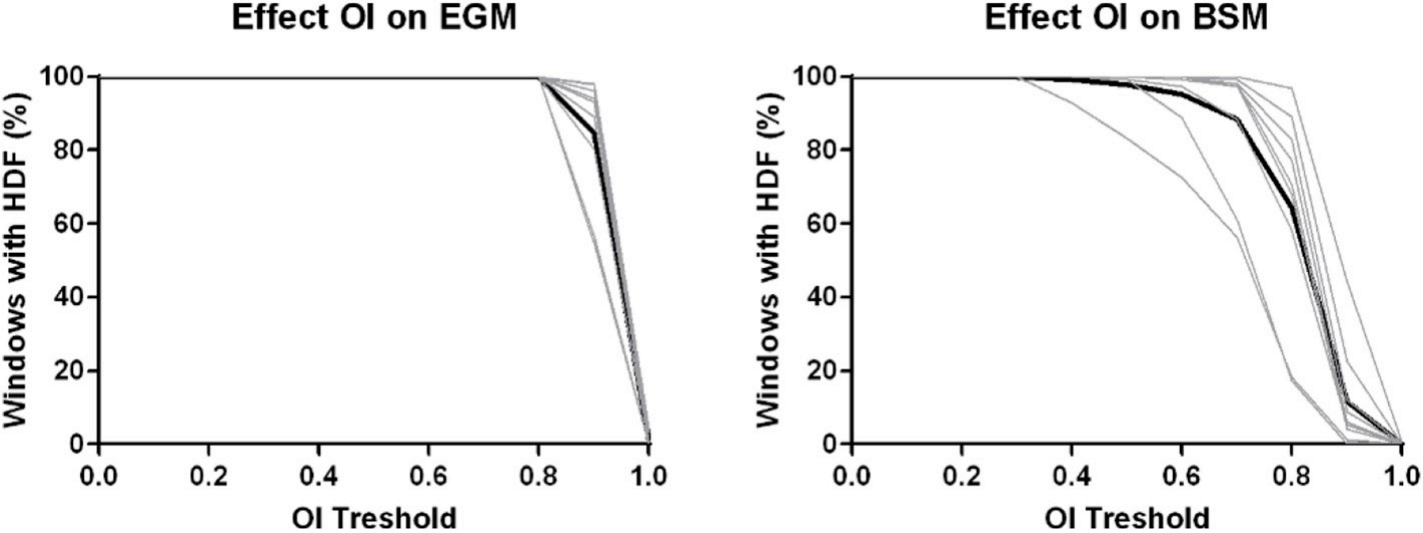
Effect of increasing the organization index (OI) threshold on the percentage of windows with a defined Highest Dominant Frequency (HDF) (excluding windows identifying harmonics or only HDFs below the OI threshold) for both virtual electrograms (VEGM, left) and body surface maps (BSM, right). Percentages are given as percentage of windows including windows for which a harmonic DF was identified. Black lines indicate the average behavior across all subjects. Grey lines indicate the behavior for individual subjects.

As studies attempting to look into frequency characteristics of AF have investigated stability of HDF areas [7,21], the effect of varying the OI threshold on the identification of HDF was investigated. Fig. 4 shows the percentage of windows that have a HDF defined for increasing OI thresholds. The percentages are based on the total number of windows investigated per subject, excluding the number of windows for which a harmonic of the main atrial rate or ventricular rate was identified. Based on the mean behavior across all patients (black lines), the percentage of windows starts decreasing from a threshold of 0.2 for BSM data. From an OI threshold above 0.76, windows with a defined HDF reduce below 50%. For VEGM data, the percentage starts dropping from a threshold of 0.8 and reaches 50% at a threshold above 0.92. The HDF behavior of the individual subjects shows that the VEGM HDF behaves more homogeneously between different subjects, whereas there is more variability amongst BSM HDF behaviors.

**Fig. 5:**
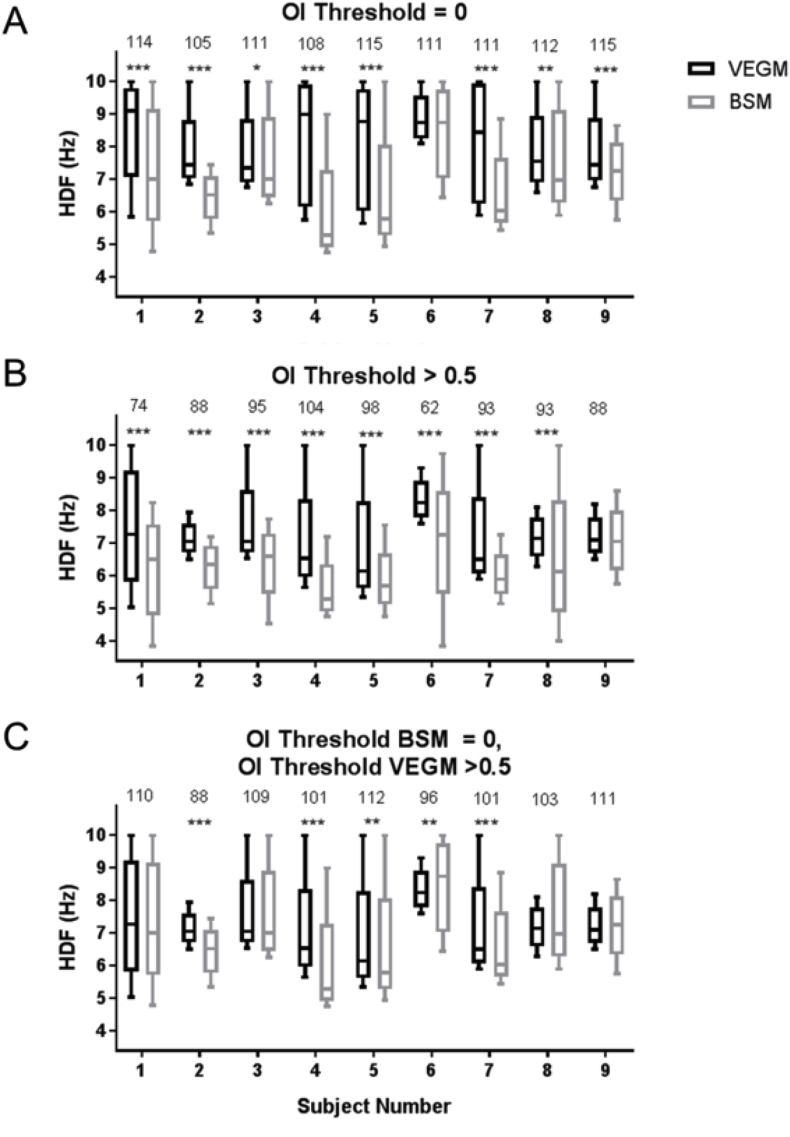
Distribution of the Highest Dominant Frequency (HDF) values on the virtual electrograms (VEGM) and body surface (BSM) for each patient (median, inter-quartile range and range are indicated). Asterisks indicate statistically significant difference (***; p < 0.001, *; p < 0.05). The number of HDF windows per bin is presented above each bin. A. No OI threshold for either group; B, OI threshold >0.5 applied to both groups; C, BSM OI threshold = 0 and VEGM OI threshold >0.5.

The HDF value on the body surface is significantly lower compared to the endocardial data in all but 1 patient (patient 6) without OI threshold (Fig. 5A), and in all but 1 patient (patient 9) after applying the OI threshold (Fig. 5B). As HDFs on the body surface might reflect activation of the entire atrium (frequencies with a high OI), rather than specifically the HDF value, the effect of applying an OI threshold to the VEGM data alone was also investigated (Fig. 5C). This showed significantly lower HDFs on the body surface for 4 subjects (2, 4, 5 and 7), and a significantly higher HDF value on the body surface for one subject (subject 6).

**Fig. 6:**
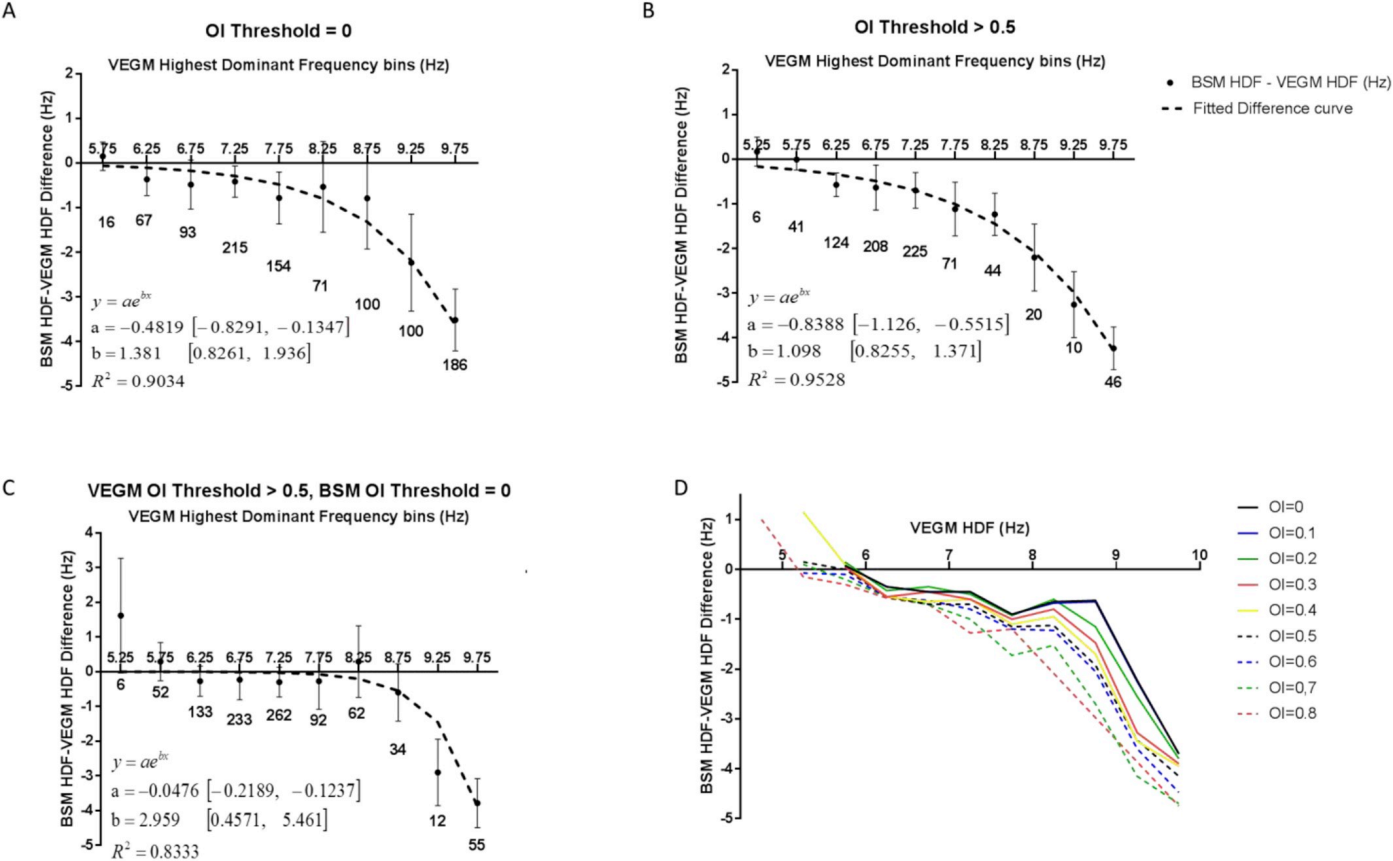
Distribution of the differences in Highest Dominant Frequency (HDF) value between body surface (BSM) and virtual electrograms (VEGM) per electrogram HDF bin. Median and spread per 0.5 Hz VEGM interval are indicated, as well as the number of windows within the interval. Parameter estimates and their 95% confidence intervals are given for a best fitting exponential curve based on the average median difference. A. No OI threshold; B; OI threshold >0.5; C. Fitted curve for VEGM OI threshold >0.5 and BSM OI = 0; D. Fitted curve for OI thresholds up to OI = 0.8.

To further analyze the behavior of HDF variation, VEGM HDF values were subtracted from their corresponding BSM HDF values. The differences are shown in Fig. 6. When increasing the OI threshold from 0 (figure 6A) to 0.5 (Fig. 6B), it appears that the VEGM HDF value reduces. Most windows have a VEGM HDF between 6.25 Hz and 7.75 Hz after thresholding, whereas most windows have a VEGM HDF between 7.25 Hz and 9.75 Hz before thresholding. However, the differences between the HDF measured on the body surface and the VEGMs remain similar. R^2^ exceeded 0.90 for all fitted curves.

The effect of the volume conductor was still visible after applying an OI threshold only to the VEGM data, albeit only strongly visible with VEGM HDF values above 8.75 Hz (Fig. 6C). From Fig. 6D, it appears that for more stable VEGM HDF values, the difference with body surface HDFs increases.

**Fig. 7:**
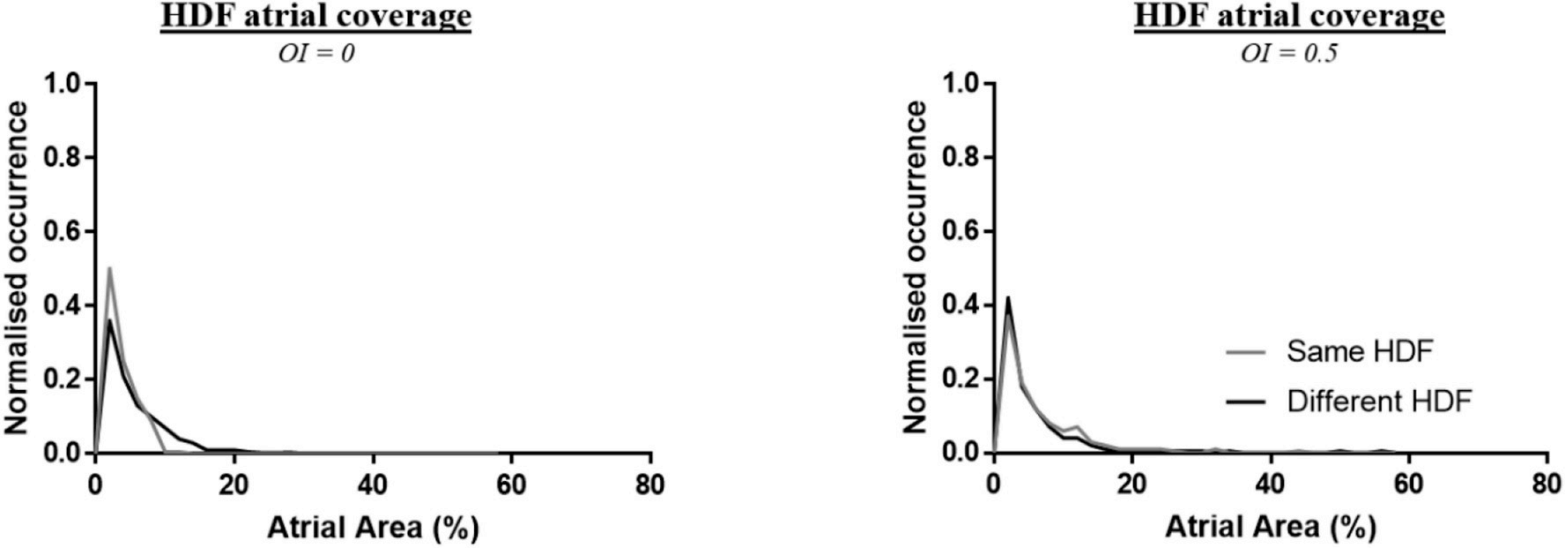
HDF atrial area distribution for VEGM and BSM HDF values with the same (grey) and different (black) HDF values on the body surface when compared against the intra-cardiac signal. HDF values were considered “Same” if the HDF of the body surface was within 0.5 Hz of the VEGM HDF value for a particular window, “Different” if otherwise. Atrial area distributions are shown for OI thresholds of 0 (left) and 0.5 (right). The Y-axis indicates the ratio of windows for which the HDF covers a specific percentage of the atria, normalized to the total number of windows.

Finally, to ensure that these findings were not affected by the atrial area occupied by the HDF region, the distribution of atrial HDF area for windows where BSM HDF was within 0.5 Hz of the VEGM HDF (“Same HDF”) was compared against that where the BSM-VEGM HDF difference exceeded 0.5 Hz (“Different HDF”). No significant difference in the distributions was found (Fig. 7, Mann-Whitney test).

## 4 Discussion

The accurate identification of regions driving AF for targeted catheter ablation has been of major interest over the last decades [[7], [8], [9],^46^]. Apart from the standardized protocol of pulmonary vein isolation [47], much attention has been paid to complex fractionated EGMs [8], focal impulses and rotors [9], as well as (highest) DF analysis [7]. Protocols for and the clinical relevance of non-invasive analysis of DF and rotors (phase analysis) are currently gaining interest [10,11].

To the best of the authors’ knowledge, the feasibility of analyzing signals taken over the complete RA and LA surfaces simultaneously has only been shown in one study [48], but without comparative BSM data. The current dataset provides the opportunity to compare measurements of unipolar VEGMs covering the entire (right and left) atrial endocardium with torso surface signals, thereby enabling us to compare the effects of different algorithms for AF source detection as well as gaining insights on the volume conductor effect using clinical data. To illustrate this, a comparison on DF and HDF behavior between BSM and VEGM data was performed.

### 4.1 DF as an indicator of AF sources

Areas of HDF might indicate local effects of atrial EGMs with highly complex organization due to wave collisions, wave breaks or other effects secondary to a mother rotor driving AF or artefacts [20]. As with other potential indicators of AF sources, DF has met with controversial results [7,21,38,48]. Originally, using bipolar point-by-point endocardial contact mapping, high frequency sites were suggested to be representative of AF sources, and a reduction of the DF gradient between the LA and RA was shown to reduce the likelihood of AF recurrence [7]. Further studies showed that areas with HDF also showed high spectral organization [50], leading to the development of the regularity [51] and organization indices [50] for assessing the dominance of the DF. By comparing (unipolar) body surface DFs with those recorded from point-by-point contact bipolar EGMs, it was found that DF areas on the body surface could be correlated to the behavior as found on the endocardial data [10]. Point-by-point DF mapping is, however, criticized for its lack in spatial resolution, and studies using unipolar VEGMs showed that DF sites were spatiotemporally unstable [39], and can wander over large distances along the endocardial wall over relatively short intervals, with the possibility of sudden disappearance and reappearance at distant locations [21]. Although these discrepancies could possibly be explained by patient-specific variations in AF behavior, variations in algorithms for determining the fundamental DF could also be a factor for these strongly varying findings. Factors influencing DF from VEGMs were investigated by Ng et al. in a series of studies [20,23,24]. This resulted in a series of recommendations, including the subtraction of ventricular activity, using signals of at least 2 s duration, application of appropriate filters, and averaging of DF over several windows. Taking these recommendations into account, the current paper further investigated the effect of stability on DF and HDF measurement on BSM and cardiac data. Investigations were performed using the OI as described previously [50].

### 4.2 Effect of DF thresholding with the organization index (OI)

An example of how OI thresholding could influence the representation of DF maps is given in Fig. 3. Applying an OI threshold of 0.5 changed the HDF location on the LA. The DF area around the mitral valve reduces after applying the OI threshold, while it increases on the atrial roof. OI thresholding could therefore have significant clinical implications in case ablation is performed based on HDF areas.

An overview of the effect of OI thresholding on HDF values is given in Fig. 4, Fig. 5. On Fig. 4, the percentage of 4 s windows with a defined HDF is plotted against the OI threshold. It is shown that HDFs can be defined from VEGM data for (almost) all windows up to an OI of 0.8, after which a steep decline occurs. From an OI of 0.9, almost none of the maps have a sufficiently stable HDF. This pattern could be reproduced for all participants in this study. The reason for the steep decrease can be understood from the definition of the OI. An OI of 1 would indicate that the complete area under the curve of the signal's spectrum would be under the HDF and its harmonics. This would indicate that the signal has a highly regular periodicity of HDF. An OI of 0.5 would mean half of the area under the curve resides under the HDF and its harmonics. It can therefore be derived that, at least for the VEGM data, for all windows under investigation, a site could be found where the HDF (and its harmonics) encompass at least half of the spectral content of the atrial signal.

Looking at the body surface data, a similar behavior of the HDF could be observed. With BSM data, however, the percentage of windows appears to reduce at lower OI thresholds (from OI = 0.2, Fig. 4) and the percentage showed larger variability between different patients compared to the VEGM data. One patient (patient 4) showed a less strong effect of OI thresholding, possibly due to a highly regular AF rate.

OI thresholding also induced changes in spatial behavior of the HDF area. On the body surface (Table 1), it can be observed that the area covered by the HDF changes significantly for most subjects after OI thresholding. Subjects 2, 4, 7 and 9, however, maintain a high number of their original HDF nodes on average, suggesting that for these subjects the change in HDF area is caused by expansion or shrinkage of the original HDF area, possibly indicating stability of the HDF area on the body surface. Subject 8, on the other hand, has no significant difference in HDF area covered (p = 0.75), but only about 30% of original HDF nodes remain after thresholding. This suggests a relocation of the HDF area due to OI thresholding, possibly indicating instability of the HDF area in this particular subject. The remaining subjects show a combination of shrinking/expansion and relocation of the HDF area.

On the atrial surface (VEGM data, Table 2), the change in HDF area is only significant for two subjects (1 and 4). The table further shows that for all except subject 7, node locations drastically changed, with less than 50% of HDF nodes without OI thresholding remaining within the HDF area after applying an OI > 0.5 Applying an OI threshold thus strongly affects the location of HDF areas, which could have significant implications for AF catheter ablation based on dominant frequency calculations. It could therefore be argued that the inclusion of a measure for the dominance of the DF and HDF peak in the spectrum could be of interest to find clinically relevant sites of HDF for targeting ablation, and optimization of this threshold needs to be sought [49]. To determine the difference in HDF between BSM and VEGM data, only OI thresholds up to 0.8 were compared, as higher OI thresholds markedly reduced the number of windows available for analysis, leading to reduced study power.

### 4.3 Volume conductor effect and discrepancies between VEGM and BSM HDF

From Fig. 5, it can be seen that, for most patients, a significant difference in HDF exists between VEGM and BSM HDF data when applying equal OI thresholds to both datasets. A window-by-window comparison showed that the HDF value on the body surface was lower than the endocardial VEGM for most windows. One of the reasons for this behavior could be the volume conductor effect, as for example seen in simulation studies on ventricular fibrillation [17] and atrial fibrillation [52]. The volume conductor can dampen high atrial rates which occur in areas of limited size on the atrium, whereas more stable atrial rates occurring over larger areas are able to ‘travel’ through the volume conductor and therefore be detected on the body surface [17,[52], [53], [54], [55], [56]]. By stabilizing the HDF only on the atrium through OI thresholding, the HDF reflects periodic signals in the VEGM data that are dominant signals within the time domain. These signals might therefore better reflect stable atrial activity, which in turn would be more likely to manifest within BSM signals. This might explain the observations comparing atrial rates with a higher OI value (and therefore a stronger presence of the particular HDF in the VEGM signal) to BSM HDF values without OI thresholding (Fig. 5C). Here, a smaller number of subjects showed lower BSM HDF values, and one subject even showed significantly higher values. Another possibility could be that high atrial frequencies occur at locations which are at a further distance from the body surface electrode locations and therefore are not visible on the body surface (e.g. the position of the appendages is closer compared to the right atrial apex when measuring with a 12-lead ECG system), although this should be accounted for by the full coverage of the torso with the BSM electrodes [29]. Further possibilities could be related to the specific algorithm used for acquiring DF and HDF values.

To further analyze a potential contribution of the volume conductor effect, BSM HDF – VEGM HDF difference distributions were compared with their corresponding VEGM HDF value. The spread of these distributions was then summarized for 0.5 Hz VEGM HDF value bins. A curve was fitted through the median values of the differences for each of these bins (Fig. 6). An exponentially decaying curve was observed, similar to the ones found in work on simulating volume conductor effects on electromyographical data [16,51,52]. Our experimental results therefore further validate the idea that the volume conductor has a (spatial) filter effect, attenuating higher frequencies found on the cardiac surface. This effect could be observed with and without the use of a DF stability threshold, although the use of a higher threshold seemed to strengthen the effect (Fig. 6D). The effect was also investigated by comparing VEGM HDF values with an OI threshold of 0.5 to BSM HDF without applying an OI threshold. This comparison still showed a volume conductor effect (Fig. 6C), albeit less pronounced. The size of the atrial HDF area also did not appear to affect this behavior, as distributions of atrial area are similar between windows in which the BSM and VEGM showed a similar HDF value (within 0.5 Hz of each other) and windows in which the BSM and VEGM showed a different HDF value (Fig. 7). One could argue as to whether the results with an OI implemented only for the VEGM data are evidence of a genuine volume conductor effect (with the highest frequencies on the atrium being dampened stronger than lower frequencies), or an effect of the body surface only showing atrial rates with a high OI threshold (which may not be indicative of AF sources [7]). Irrespective of the reason, it shows that care should be taken with determining AF characteristics based solely on body surface signals [4,10,11].

Previous results have shown that, based on the relative distance from the heart to the torso, a strong volume conductor effect could occur at frequencies of 5–10 Hz [57]. As endocardial HDF within this frequency range might present low power, this HDF might not consistently be apparent on the body surface. Early simulations focused on establishing a biophysical background for the implementation of advanced forward and inverse solutions also observed similar effects [12, 58]. As non-invasive analysis of AF could form an intermediate between invasive EP studies and non-invasive inverse problem analysis, these effects have to be taken into consideration when deriving information of atrial fibrillation sources based solely on body surface signals.

## 5 Study limitations

Although 5-min recordings were considered in this work, larger patient cohorts would aid in strengthening the current analysis. EGMs were estimated using a commercialized inverse solution, for which the temporal correlation with contact unipolar mappings is, on average, only reasonable [39, 59]. DF comparisons between the inversely-solved VEGMs and contact intra-cardiac EGMs however appear to correlate strongly [39]. Lastly, no attempt was made to determine HDF sites on the cardiac surface from body surface signals using an inverse solution. A recent study has however shown atrial DFs can be accurately mapped from BSM using a frequency-based inverse solution [60]. Applying an inverse solution might also help explain the behavior of the OI index in the BSM signals. It might be that high regularity cannot be expected on the BSM due to a weighted summation of contributions from atrial regions, which can have clearly distinct DF values due to DF gradients [7].

A problem with the detection of (highest) DFs based on frequency power is the potential issue of identifying harmonics as the DF. In this paper, we suggest the use of an F-test to determine the significance of the ratio between the power of the DF and its neighboring frequencies. This approach has been used previously in determining objective responses in electro-encephalography (EEG) studies [36], but not yet adopted in AF DF analysis. Although this approach might not fully guarantee avoiding detection of harmonics, it provides additional verification of the contribution of the DF peak within the spectrum, and therefore the likelihood of the DF peak being genuine compared to the power of other frequencies within the DF range. Higher frequencies might also need to be included in the DF spectrum (as, e.g. in Refs. [39,55]) to avoid saturation of HDFs towards the frequency range limits. Further research is needed in determining the optimal frequency range for detection of DF and HDFs on VEGM and BSM data.

## 6 Conclusion

This paper investigated the effect of the volume conductor on DF representations of human atrial fibrillation collected from body surface signals (BSMs) and compared with simultaneously recorded virtual intra-cardiac electrograms (VEGMs). The impact of OI thresholding on the identification of HDF areas on both VEGM and BSM data was also demonstrated. We show that, to derive clinically relevant information about AF sources from non-invasive body surface data, the physical background of the volume conductor effect should be taken into consideration. The results of our work provide real-world experimental confirmation of the low-pass effect of the volume conductor postulated previously by simulation studies. For the identification of HDF areas, it is of interest to take into account the OI. As highest DF regions may not have the highest OI, a suitable organization threshold could help in locating sources of AF activation with increased confidence.

## 7 Summary

In this work, we characterize and validate the volume conductor effect on the representation of atrial fibrillation (AF) dominant frequencies (DF) on the body surface using data obtained from patients with AF. DF behavior was analyzed on 5 min of AF data collected simultaneously from a 131-electrode body surface mapping system and from both atria using non-contact catheters. DFs were identified as the frequency with highest power between 4 and 10 Hz in 4-s windows. Areas with highest dominant frequency (HDF) values were identified as areas which had a DF within 0.25 Hz of the highest value found on both atria and the body surface separately. The effect of DF organization thresholding was investigated using the organization index (OI).

Applying a DF organization threshold can significantly alter the representation of DF on the atria and body surface. With increasing OI threshold, the number of VEGMs displaying a stable DF reduced, occasionally leading to different HDF areas being identified. Alongside this behavior, HDF values significantly decreased in all patients when OI thresholding was applied. These results indicate that some form of assessing DF stability should therefore be considered in order to indicate atrial fibrillation ablation targets.

Comparing HDF values on the atria and body surface in 0.5 Hz bins clearly identified the effect of the volume conductor, with body surface HDF values decaying exponentially with increasing atrial HDF values, agreeing with previous simulation studies. This volume conductor effect was independent of OI thresholding. Volume conductor effects should therefore be taken into consideration when AF behavior is analyzed solely using body surface signals.

## Funding sources

The work was funded by the National Institute for Health Research (NIHR) Leicester Cardiovascular Biomedical Research Centre, The British Heart Foundation (BHF Project Grant no. PG/18/33/33780) and the East Midlands Pacemaker Fund. J.S. and T.P.A. were supported by The National Council for Scientific and Technological Development (CNPq) of Brazil, and the Co-ordination for the Improvement of Higher Education Personnel (CAPES).

## Conflicts of interest statement

G.A. Ng has received research fellowship from St. Jude Medical and speaker fees and honoraria from Biosense Webster.

The other authors declare that the research was conducted in the absence of any commercial or financial relationships that could be construed as a potential conflict of interest.
